# Effect of Vitamin D supplementation on synovial tissue volume and subchondral bone marrow lesion volume in symptomatic knee osteoarthritis

**DOI:** 10.1186/s12891-019-2424-4

**Published:** 2019-02-14

**Authors:** Thomas A. Perry, Matthew J. Parkes, Richard Hodgson, David T. Felson, Terence W. O’Neill, Nigel K. Arden

**Affiliations:** 1 Arthritis Research UK Centre for Epidemiology, Faculty of Biology, Medicine and Health, Manchester Academic Health Science Centre, Research in Osteoarthritis Manchester (ROAM), Division of Musculoskeletal and Dermatological Sciences, The University of Manchester, School of Biological Sciences, Stopford Building, Oxford Road, Manchester, M13 9PT UK; 2 NIHR Manchester Biomedical Research Centre, Manchester Academic Health Science Centre, Manchester University NHS Foundation Trust, Manchester, UK; 3 Centre for Imaging Sciences, Institute of Population Health, University of Manchester, Manchester, UK; 4 Department of Rheumatology, Salford Royal NHS Foundation Trust, Salford, UK; 5 Clinical Epidemiology Research and Training Unit Boston University School of Medicine, Boston, MA USA; 6 Nuffield Department of Orthopaedics, Rheumatology and Musculoskeletal Sciences, University of Oxford, Oxford, UK; 7 Medical School, University of Sydney, Sydney, Australia

**Keywords:** Vitamin D, Osteoarthritis, Synovitis, Bone marrow lesions (BMLs)

## Abstract

**Background:**

Data from a recent clinical trial of vitamin D therapy in knee OA suggests that, compared to placebo, vitamin D therapy may be associated with a reduction in effusion-synovitis. Our aim was, using contrast-enhanced (CE) magnetic resonance imaging (MRI), to examine the effect of vitamin D therapy on synovial tissue volume (STV) and also subchondral bone marrow lesion (BML) volume in men and women with symptomatic knee OA.

**Methods:**

Data was acquired from participants who took part in a randomised placebo-controlled trial (UK VIDEO) investigating the effect of vitamin D therapy (800 IU cholecalciferol daily) on radiographic joint space narrowing. A subsample had serial CE MRI scans acquired during the trial. Subjects with serial images were assessed (*N* = 50) for STV and subchondral BML volume. The difference in the mean change from baseline in these structural outcomes between intervention and placebo groups was assessed using random-effects modelling.

**Results:**

The mean age of the 50 subjects (24 active group, 26 placebo group) who contributed data to the analysis was 63.3 years (SD 6.5) and 74% were female. There was no significant difference at 2 years follow-up between the vitamin D and placebo groups in the mean change from baseline for STV (93.9 mm^3^, 95% CI -1605.0 to 1792.7) and subchondral BML volume (− 313.5 mm^3^, 95% CI -4244.7 to 3617.7).

**Conclusions:**

Vitamin D supplementation does not appear to have an effect on synovitis or BML volume in patients with symptomatic knee OA.

**Trial registration:**

VIDEO was registered with EudraCT: ref. 2004-000169-37. The protocol for the trial can be accessed at https://www.ctu.mrc.ac.uk/studies/all-studies/v/video/

**Electronic supplementary material:**

The online version of this article (10.1186/s12891-019-2424-4) contains supplementary material, which is available to authorized users.

## Background

Vitamin D has well recognised biological effects on bone and cartilage [1]. Vitamin D may act also as a moderator of innate and adaptive immunity [2] given the expression of the vitamin D receptor (VDR) by circulating macrophages, monocytes and dendritic cells [1, 2] suggesting that serum vitamin D status may be clinically relevant in terms of inflammation.

There is evidence from prospective studies that low serum vitamin D may be linked with structural change in the knee. Data from the Framingham study suggests that low serum vitamin D is associated with increased odds of radiographic knee OA progression after adjustment for age, sex, BMI, physical activity and knee injury [3]. Data from the Osteoarthritis Initiative (OAI) study also supports an association between low serum vitamin D and an increased risk of radiographic progression in symptomatic knee OA [4]. Data from a number of randomised clinical trials, however, has suggested no effect of vitamin D therapy either on pain or joint space narrowing assessed on plain radiographs [5, 6].

In a trial of men and women with symptomatic knee OA, however, treated with either vitamin D (50,000 IU/monthly) or placebo over a 2 year period, and who had non-contrast enhanced MRIs performed at baseline and follow-up, there was evidence that compared to placebo, vitamin D therapy was linked with a smaller increase in effusion-synovitis [7]. Effusion-synovitis does not, however, distinguish between the presence of true synovitis (thickening of synovial tissue) and effusion for which contrast-enhanced scans allow for more sensitive differentiation [8]. Data from two trials in which MRI scans were performed did not suggest any effect of vitamin D therapy on bone marrow lesion size [6, 9].

Using data from a randomised placebo-controlled trial of vitamin D therapy in knee OA we looked at the impact of vitamin D therapy on synovial tissue volume (STV) and subchondral BMLs in a subsample of participants who had CE MRI scans performed. We hypothesised that vitamin D therapy would be linked with a reduction in STV.

## Methods

### Subjects

Subjects who took part in this analysis were participants in the Southampton (UK) arm of the Vitamin D in Osteoarthritis (VIDEO) study [5] which was a 3-year double-blind, randomised, placebo-controlled clinical trial involving five UK NHS centres (Oxford, Manchester, Southampton, Norwich, Newcastle). The aim of the VIDEO study was to investigate the efficacy of vitamin D therapy on radiographic assessed joint space narrowing in symptomatic knee OA patients.

### VIDEO trial

The detailed VIDEO study methods have been described previously [5]. In brief, participants were recruited from both primary and secondary care, had a Kellgren and Lawrence (K&L) score [10] of 1–4 in the medial tibio-femoral knee compartment, joint space width (JSW) > 1 mm and knee pain for most days of the previous month. Patients who satisfied the inclusion criteria and who consented to take part were subsequently randomised, with stratification by recruitment centre, using computer-generated number allocation to receive either oral vitamin D of 800 IU/daily or matching placebo in a 1:1 ratio. There was no requirement for participants to have a specific level of serum vitamin D in order to be eligible for the study. The main outcomes were rate of joint space narrowing as assessed using plain radiographs and also knee symptoms as assessed using the Western Ontario and McMaster Universities Arthritis Index (WOMAC) questionnaire with each item scored on a visual analogue scale (VAS) (0–100 mm range, 100 = worse pain/disability).

Blood samples were taken at baseline and were repeated at 12 and 36 months follow-up to measure serum vitamin D_3_ (25-OH-D_3_). At the Southampton centre, serial MRIs were performed at baseline and at 12-monthly intervals. Subjects (*N* = 50) were included in the current analysis if they had; (i) a baseline sagittal T_1_-weighted (T_1_-w) fat suppressed (FS) contrast-enhanced (CE) scan, (ii) a minimum of 1 follow-up sagittal T_1_-w FS CE scan over the first 2 years of study using the same index knee and (iii) at least an axial proton density weighted FS and/or coronal STIR scan at the corresponding visits.

### Magnetic resonance imaging

Images were acquired using a 1.5 T MRI scanner (Signa (GE Healthcare)) and a dedicated phased-array knee coil [11]. Sagittal post-contrast T_1_-w FS (TR = 600–800 ms, TE = 12.5–16.2 ms, acquisition matrix 256 × 160, slice gap = 0.6 mm, slice thickness = 3 mm), axial proton density weighted (PD-w) FS (TR = 3800–4820 ms, TE = 31.2–32.5 ms, matrix 256 × 192, slice gap = 0.2 mm, slice thickness = 4 mm) and coronal short tau inversion recovery (STIR) (TR = 3000–4760 ms, TE = 46.1–56.9 ms, matrix 256 × 192, slice gap = 0.3 mm, slice thickness = 3 mm) sequences were obtained at baseline and follow-up visits. Patients were positioned supine for scanning. An intravenous injection of gadodiamide (0.2 mL/kg body weight (Omniscan, GE Healthcare)) [11] was administered 3 min prior to the acquisition of the first CE scan; with all scans acquired within 11 min of contrast administration.

### Quantitative assessment of volume

A single reader performed segmentation of the sagittal CE scans on both the baseline and follow-up scans (TP). All results were based upon measurements taken on sagittal T_1_-w FS CE images though segmentation was guided by complimentary axial and/or coronal sequences. The segmenter was blinded to treatment allocation. Patient identifiers were randomised: within each patient’s image set, visit order was randomised. Randomisation was completed by a separate member of the research team who took no part in the segmentation of images. In order to optimise our ability to detect change in volume, images were assessed in groups with all visits being segmented before moving onto the next patient – a method previously described by Felson et al [12]. Segmentation was performed on a DELL desktop computer (Intel Core 2 Duo, Dell Inc., Round Rock, TX) running a Microsoft Windows operating system (Microsoft Inc., Redmond, WA).

### Quantitative measurement of synovial tissue volume (STV)

STV was assessed across the whole knee joint using a semi-automated approach [13]. STV and popliteal cysts were segmented to the point where the gastrocnemius tendon was adjacent to the semi-membranosus tendon. The segmenter manually cropped images across all visits for a given participant (when appropriate) before moving onto the next to control for differences in knee position within the field of view.

### Quantitative assessment of subchondral bone marrow lesion (BML) volume

Bone marrow lesions within the subchondral bone were measured across the whole knee joint and were defined as ill-defined hyperintensities on post-contrast fat suppressed MRI. Subchondral BMLs were included for volumetric assessment if present on ≥2 consecutive slices; in accordance with previous studies [14, 15]. Hyperintensities within osteophytes that were not extending beyond the margins of osteophytes into the subchondral bone were not included. Regions of well-defined high signal (in relation to oedema-like signal) on post-contrast scans within subchondral BMLs, which other groups have associated with subchondral cysts [16], were included in the total volume measurement. Intra- and inter-observer agreement for both BML volume and STV has been reported previously and found to be excellent [13].

### Statistical analysis

Baseline characteristics were summarised using means and standard deviations (SD) for normally distributed variables. Frequencies were used to describe categorical variables. Paired t-tests were used to compare change in mean serum vitamin D between baseline and at 12 months follow-up.

To determine the impact of treatment on structural outcomes we used random-effects multiple linear panel regression, with change in synovial tissue volume from baseline (or subchondral BML volume) as the outcome, treatment (a categorical variable whereby a code of 1 = placebo group, and 2 = vitamin D group) and visit (coded as either 1 = year 1 or 2 = year 2) as the predictor variables. The treatment term in this model therefore tests whether the change in STV/subchondral BML volume differed between treatment groups. A visit-by-treatment interaction effect was also included to assess if the between-groups treatment effect differed according to the length of follow-up (either 1 year or 2 years).The model was adjusted for synovial tissue volume (or subchondral BML volume) at baseline, age and gender. The correlation of observations within individuals was addressed by setting patient identifier as a panel variable. All statistical analysis was completed using Stata/IC version 14.0 (Stata Corp., College Station, Texas, USA). A two-sided *P* value of 0.05 was considered statistically significant. In a sensitivity analysis, we performed a one sided test (H_0_ ≥ 0, H_1_ ≤ 0) to increase the power to detect a difference between vitamin D and placebo groups at 2 years follow-up assuming that vitamin D would lead to a reduction in STV and subchondral BML volume.

## Results

### Subjects

As part of the VIDEO trial, 174 subjects were recruited at Southampton. Of these, 50 had serial CE MRIs including one baseline and at least another CE scan during the first 2 years of the study. Of these, 24 were assigned to vitamin D therapy and 26 to placebo. Table 1 shows baseline clinical data and, radiographic and MRI characteristics of these subjects. Of the 50 patients who contributed data, the treatment and placebo groups appeared well matched. Reasons for excluding the remaining 124 participants included: no MRI data, an absence of baseline MRI (particularly CE-MRI) or follow-up MRI.

**Table 1:** Subject characteristics at baseline by treatment group

Variable	Vitamin D Therapy (*N* = 24)	Placebo (*N* = 26)
Age (years)	63.0 (5.8)	63.6 (7.2)
Females, n (%)	17 (70.8)	20 (76.9)
Index knee, n (% Right)	14 (58.3)	15 (57.7)
Height (m^2^)	1.68 (0.09)	1.68 (0.10)
Weight (kg)	79.2 (10.1)	82.1 (17.0)
Body mass index (BMI) (kg/m^2^)	28.2 (4.0)	29.2 (5.6)
Serum vitamin D_3_ (μg/L)	26.6 (8.5)	25.3 (8.7)
Vitamin D deficient (< 20 μg/L), n (%)	6 (25)	7 (27)
Worst Kellgren–Lawrence (KL) grade^a^ (medial/lateral) in index knee		
Grade 1, n (%)	6 (25.0)	2 (7.7)
Grade 2, n (%)	8 (33.3)	14 (53.9)
Grade 3, n (%)	8 (33.3)	8 (30.8)
Grade 4, n (%)	2 (8.3)	2 (7.7)
WOMAC^b^ pain score	29.4 (15.6)	34.3 (19.5)
WOMAC stiffness score	45.3 (22.9)	50.4 (22.4)
WOMAC function score	33.0 (20.4)	38.6 (21.2)
WOMAC total score	33.3 (18.8)	38.7 (19.9)
Synovial tissue volume (mm^3^)	11,879.8 (7112.3)	12,646.6 (5305.5)
Total Subchondral BML volume (mm^3^)	7159.1 (7739.2)	5765.6 (8839.8)

Results are shown as mean (SD) or frequencies (%) unless stated otherwise

^a^ Worst KL score was defined as the maximum score across the medial and lateral sites for the index knee

^b^ Western Ontario and McMaster Universities Osteoarthritis Index (WOMAC) questionnaire using a visual analogue scale (VAS) to score pain, function, stiffness and total (sum of score outcomes) from 0 to 100 (0 = no pain / disability, 100 = high pain / disability)

STV and subchondral BML volumes of the index knee were missing for a total of 6 patients (12.0%) at year 1 (placebo = 3, vitamin D = 3) and 19 patients (38.0%) at year 2 (placebo = 9, vitamin D = 10). Missingness was caused in part by the exclusion of one visit from 1 patient due to MRI parameters which were beyond the normal scope for the sequence type. The remaining missing observations were due to an absence of imaging data due to technical and/or logistic reasons. Missingness of MRI data did not vary by treatment arm. There were no differences in the baseline characteristics between those 50 subjects used in the analysis and the remaining Southampton study population (*N* = 124) other than that the mean baseline serum vitamin D which was significantly higher in those who contributed data to the analysis reported here compared to those who did not; see Additional file 1: Table S1.

### Effect of intervention on Vitamin D levels

Of the 50 patients who contributed data, serum vitamin D_3_ levels increased significantly in the vitamin D group from a mean of 27.5 μg/L (SD 8.3) at baseline to 32.6 μg/L (SD 7.2) at 12 months follow-up; mean change 5.2 μg/L (SD 5.0) (*P* < 0.001). In contrast, serum vitamin D_3_ levels decreased significantly in the placebo group from a mean of 25.3 μg/L (SD 8.7) at baseline to 22.7 μg/L (SD 8.5) at 12 months follow-up; mean change was − 2.5 μg/L (SD 6.1) (*P* = 0.05). Further, the number of subjects with vitamin D deficiency (< 20 μg/L) [5, 17] fell from 25.0% (*N* = 6) to 4.2% (*N* = 1) in the vitamin D intervention group at 12 months follow-up though increased in the placebo group from 26.9% (*N* = 7) to 34.6% (*N* = 9).

### Effect of intervention on STV and subchondral BML volume

After adjusting for baseline values of the outcome, age and gender there was no significant difference at year 1 in the mean change in total synovial tissue volume from baseline between the vitamin D and placebo groups (− 54.6 mm^3^, 95% CI -1537.8 to 1428.5); see Table 2. Looking within groups, compared to baseline, there was a small, non-significant decrease in total STV in those on vitamin D (− 364.3 mm^3^, 95% CI -1439.1 to 710.6) and also in the placebo group (− 309.6 mm^3^, 95% CI -1330.6 to 711.4). Further, there was no significant difference in the mean change from baseline in total synovial tissue volume between groups at 2 years (93.9 mm^3^, 95% CI -1605.0 to 1792.7); with both the vitamin D group (155.4 mm^3^, 95% CI -1097.3 to 1408.0) and placebo group (61.5 mm^3^, 95% CI -1085.6 to 1208.6) showing a small, non-significant increase in total STV compared to baseline. The visit-by-treatment interaction was non-significant.

**Table 2:** Treatment effect estimates for synovial tissue volume for vitamin D and placebo groups

	Change from baseline. Mean (mm^3^), 95% CI	
	Year 1	Year 2
Vitamin D Therapy	−364.3	155.4
	(−1439.1 to 710.6)	(−1097.3 to 1408.0)
Placebo	−309.6	61.5
	(− 1330.6 to 711.4)	(−1085.6 to 1208.6)
Adjusted^a^ Mean Difference between groups	−54.6	93.9
	(−1537.8 to 1428.5)	(−1605.0 to 1792.7)

All results presented with 95% confidence intervals (CI)

^a^ Adjusted mean difference between vitamin D and placebo groups (*N* = 50) at follow-up generated from random-effects models; adjusted for baseline synovial tissue volume, age and sex

After adjusting for baseline values of total subchondral BML volume, age and gender, there was no significant difference at year 1 in the mean change in total subchondral BML volume from baseline between the vitamin D and placebo groups; 2175.7 mm^3^ (95% CI -1263.8 to 5615.1); see Table 3. In the placebo arm, total subchondral BML volume decreased but not significantly from baseline by a mean of − 506.7 mm^3^ (95% CI -2870.3 to 1856.9), while in the vitamin D arm, BML volume increased but not significantly by a mean of 1669.0 mm^3^ (95% CI -827.9 to 4165.9). Further, there was no significant difference at year 2 in the mean change in total subchondral BML volume from baseline; − 313.5 mm^3^ (95% CI -4244.7 to 3617.7). In those on vitamin D, there was a small, non-significant decrease in BML volume from baseline (− 506.9 mm^3^, 95% CI -3395.6 to 2381.9) and also in the placebo group (− 193.4 mm^3^, 95% CI -2845.7 to 2459.0). The visit-by-treatment interaction was non-significant.

**Table 3:** Treatment effect estimates for subchondral BML volume for vitamin D and placebo groups

	Change from baseline. Mean (mm^3^), 95% CI	
	Year 1	Year 2
Vitamin D Therapy	1669.0	−506.9
	(−827.9 to 4165.9)	(−3395.6 to 2381.9)
Placebo	−506.7	− 193.4
	(− 2870.3 to 1856.9)	(−2845.7 to 2459.0)
Adjusted^a^ Mean Difference between groups	2175.7	−313.5
	(−1263.8 to 5615.1)	(−4244.7 to 3617.7)

All results presented with 95% confidence intervals (CI)

^a^ Adjusted mean difference between vitamin D and placebo groups (*N* = 50) at follow-up generated from random-effects models; adjusted for baseline subchondral BML volume, age and sex

In sensitivity analysis, further adjustment for baseline body mass index (BMI) and baseline K&L grade did not affect the between group difference for either STV or subchondral BML volume at 2 years follow-up (see Additional file 2: Table S2). Using a one-sided test, there was no significant difference between vitamin D and placebo groups at 2 years follow-up in mean change from baseline for STV (93.9 mm^3^, 95% one-sided CI 1190.1, one-sided *P* = 0.47) or subchondral BML volume (− 313.5 mm^3^, 95% one-sided CI 5062.1, one-sided *P* = 0.9).

## Discussion

In this analysis, compared to those taking placebo, vitamin D therapy (800 IU/daily) for 2 years had no significant effect on reducing synovial tissue volume or subchondral BML volume among those with symptomatic knee OA.

Two published trials have looked at MRI outcomes following vitamin D intervention in patients with symptomatic knee OA, though in only one of these, the Australian VIDEO study, did the authors look at synovitis [7]. In this trial participants were randomised to vitamin D therapy (50,000 IU/monthly) or placebo. In a post-hoc analysis the authors reported a mean between-group difference in effusion-synovitis of − 1.94 mL (95% CI -3.54 to − 0.33) at 2-years follow-up with the mean increase in effusion-synovitis volume over follow-up significantly less in the vitamin D therapy group (0.26 mL, 95% CI − 0.82 to 1.34) compared to the placebo group (2.20 mL, 95% CI 1.01 to 3.38) [7]. The data however, were obtained using non-contrast images – such images are less sensitive in distinguishing effusion from underlying synovitis. In the Australian VIDEO study there was evidence to suggest that the effect on effusion-synovitis may have been more marked in those with low vitamin D levels; i.e. those who were consistently sufficient (> 50 nmol/L) at both 3 and 24 months follow-up had a significantly smaller increase in mean effusion-synovitis volume compared to those who were consistently insufficient (≤50 nmol/L) (− 2.5 mL, 95% CI -4.7 to − 0.2) [18]. Because of relatively small numbers of those with low vitamin D levels, we were unable to confirm or refute the findings of the Australian study in terms of a potential moderating effect of vitamin D status on treatment effect.

Our data are in keeping with previous studies showing no significant change in BMLs in response to vitamin D therapy. In a 24 month randomised trial of vitamin D therapy (2000 IU/daily), among those aged ≥45 years with symptomatic knee (*N* = 146) BML volume was assessed at baseline and follow up at the femur and tibia using sagittal intermediate-weighted (IW) FS scans [6]. There was no evidence of a significant difference in femoral BML or tibial BML volume between treatment arms at 2 years follow-up [6]. In the Australian VIDEO trial after 24 months there was a mean decrease in tibiofemoral BML score in the vitamin D group and a mean increase in the placebo group; the between group difference at 2 years just failed to reach conventional levels of statistical significance (mean difference: -0.5, 95% CI -0.9 to 0.0) [9]. Our data extend these findings and includes also BMLs from the whole joint including the tibiofemoral and patellofemoral joints.

Our study had a number of strengths; the main being that CE MRIs were acquired allowing more accurate assessment of synovitis. There are though some limitations to consider in interpreting our data. One of the main limitations was that the number of subjects who took part in the analysis was relatively small (*N* = 50) and the study therefore had limited power to determine a difference in structural outcomes between those on or not on vitamin D therapy. The magnitude of the observed effect on STV, however, was small and in the opposite direction to that which would have been expected if vitamin D therapy had a beneficial effect on reducing synovitis. Furthermore, evidence from one-sided testing also showed no suggestive effect. In our study there was no requirement for subjects to have specific vitamin D levels and many subjects in the study were vitamin D sufficient at baseline. It is possible that inclusion of these subjects may have reduced the chance of finding an effect of vitamin D on structural change for which further studies would be required. Not all subjects recruited to the study in Southampton had MRIs performed and it is possible that the structural parameters including synovitis may have differed in those who did and did not participate. Baseline characteristics, however, of those participants who were included in the analysis were not significantly different from those also recruited to the Southampton site who did not take part, apart from baseline serum vitamin D; with those studied having greater serum vitamin D. It seems unlikely however, that any such difference would impact on the results which were based on an internal comparison of those who took part in the analysis. We included data from only one of the participating VIDEO centres, however, in the study randomisation was stratified by centre which would give confidence that whilst the sample was small, randomisation was maintained at the Southampton site. Finally in our study subjects in the intervention arm received 800 IU vitamin D per day and were followed for up to 2 years, and we cannot exclude an effect of a higher dose and given over a longer period of time.

## Conclusions

Treatment by vitamin D supplementation, at a specified dose of 800 IU/day for 2 years, did not lead to a significant reduction in synovial tissue volume or subchondral BMLs in those with symptomatic knee OA over 2 years. The data suggest that vitamin D therapy may not have an effect on structural change in men and women with symptomatic knee OA.

## Additional files

Additional file 1: **Table S1** Subject characteristics of those included and those not included in the analysis. (DOCX 18 kb)

Additional file 2: **Table S2** Treatment effect estimates for vitamin D and placebo groups in multivariate analysis. (DOCX 16 kb)

## Abbreviations

BMI: body mass index
BML: bone marrow lesion
CE: Contrast-enhanced
FS: fat suppressed
IQR: interquartile ranges
JSN: joint space narrowing
K&L: Kellgren and Lawrence
MRI: magnetic resonance imaging
OAI: Osteoarthritis Initiative
SD: standard deviation
STIR: short tau inversion recovery
STV: synovial tissue volume
T_1_-w: T_1_-weighted
VAS: visual analogue scale
VDR: vitamin D receptor
WOMAC: Western Ontario and McMaster Universities Arthritis Index
