# Supplementary material for: Effect of Vitamin D supplementation on synovial tissue volume and subchondral bone marrow lesion volume in symptomatic knee osteoarthritis
Source: BMC Musculoskelet Disord. 2019 Feb 14;20:76. doi: 10.1186/s12891-019-2424-4 (PMC6376763; doi:10.1186/s12891-019-2424-4)
Supplement: Supplementary file 1 — Table S1 Subject characteristics of those included and those not included in the analysis. (DOCX 18 kb) [file 12891_2019_2424_MOESM1_ESM.docx]

**Additional file 1: Table S1:** Subject characteristics of those included and those not included in the analysis.

| Variable | Study sample (N = 50) | | | Recruited though not included in the analysis (N = 124) | P Values |
| --- | --- | --- | --- | --- | --- |
| Age (years) | 63.3 (6.5) | | | 65.0 (8.4) | 0.22 |
| Females, n (%) | 37 (74) | | | 77 (62.1) | 0.14 |
| Index knee, n (% Right) | 29 (58) | | | 67 (54.0) | 0.63 |
| Height (m^2^) | 1.7 (0.1) | | | 1.7 (0.1) | 0.67 |
| Weight (kg) | 80.7 (14.1) | | | 82.4 (13.8) | 0.49 |
| Body mass index (BMI) (kg/m^2^) | 28.7 (4.9) | | | 29.5 (4.9) | 0.35 |
| Serum vitamin D_3_ (µg/L) | 25.9 (8.5) | | | 19.6 (8.4) | < 0.001 |
| Vitamin D deficient (<20 µg/L), n (%) | 13 (26) | | | 70 (56.5) | < 0.001 |
| Worst Kellgren–Lawrence grade^≠^ (medial/lateral) in index knee | | | | |  |
| Grade 1, n (%) | | 8 (16.0) | 23 (18.6) | | 0.69 |
| Grade 2, n (%) | | 22 (44.0) | 54 (43.6) | | 0.96 |
| Grade 3, n (%) | | 16 (32.0) | 38 (30.7) | | 0.86 |
| Grade 4, n (%) | | 4 (8.0) | 9 (7.3) | | 0.87 |
| WOMAC^†^ pain score | | 32.0 (17.7) | 30.1 (17.7) | | 0.53 |
| WOMAC stiffness score | | 48.0 (22.5) | 43.4 (23.5) | | 0.24 |
| WOMAC function score | | 35.9 (20.8) | 33.1 (20.2) | | 0.42 |
| WOMAC total score | | 36.1 (19.3) | 33.4 (18.7) | | 0.40 |
| Results are shown as mean (SD) or frequencies (%) unless stated otherwise.  ^≠^ Worst KL score was defined as the maximum score across the medial and lateral sites for the index knee.  ^†^ Western Ontario and McMaster Universities Osteoarthritis Index (WOMAC) questionnaire using a visual analogue scale (VAS) to score pain, function, stiffness and total (sum of score outcomes) from 0 to 100 (0 = no pain / disability, 100 = high pain / disability).  Two-sample t tests were used to compare groups. | | | | | |
