# Supplementary material for: Effect of Vitamin D supplementation on synovial tissue volume and subchondral bone marrow lesion volume in symptomatic knee osteoarthritis
Source: BMC Musculoskelet Disord. 2019 Feb 14;20:76. doi: 10.1186/s12891-019-2424-4 (PMC6376763; doi:10.1186/s12891-019-2424-4)
Supplement: Supplementary file 2 — Table S2 Treatment effect estimates for vitamin D and placebo groups in multivariate analysis. (DOCX 16 kb) [file 12891_2019_2424_MOESM2_ESM.docx]

**Additional file 2: Table S2:** Treatment effect estimates for vitamin D and placebo groups in multivariate analysis.

|  | Change from baseline. Mean (mm^3^), 95% CI | | | | | |
| --- | --- | --- | --- | --- | --- | --- |
|  | **Year 2** | | | | | |
| Variable | **Multivariate ^a^** |  | **Multivariate ^b^** |  | **Multivariate ^c^** |  |
| Total Synovial Tissue Volume | 131.8  (-1552.4 to 1816.0) | 0.88 | 91.9  (-1626.0 to 1809.7) | 0.92 | 140.1  (-1563.0 to 1843.2) | 0.87 |
| Total Subchondral BML Volume | -247.5  (-4152.1 to 3657.1) | 0.90 | -446.7  (-4388.7 to 3495.3) | 0.82 | -87.2  (-3957.9 to 3783.5) | 0.97 |
| Abbreviations: BMI, Body Mass Index (kg/m^2^) and BML, Bone Marrow Lesion.  All results presented with confidence intervals (95% CI) and P values.  ^≠^ Adjusted mean difference between vitamin D and placebo groups (N = 50) at follow-up generated from random-effects models.  ^a^ Adjustment for baseline total synovial tissue (or baseline subchondral BML volume) only.  ^b^ Adjustment for baseline total synovial tissue volume (or baseline subchondral BML volume), age, sex and BMI.  ^c^ Adjustment for baseline total synovial tissue volume (or baseline subchondral BML volume), age, sex, BMI and baseline K&L score. | | | | | | |
